# Association Between Polygenic Risk Scores and Treatment Response to Antidepressants, Benzodiazepines, and Antihistamines in Anxiety and Depression

**DOI:** 10.1016/j.bpsgos.2025.100470

**Published:** 2025-02-18

**Authors:** Amelie Markant, Fara Tabrizi, Hampus Grönvall, Doug Speed, Fredrik Åhs

**Affiliations:** a Department of Psychology, University of Bremen, Bremen, Germany; b Department of Psychology and Social Work, Mid Sweden University, Östersund, Sweden; c Center for Quantitative Genetics and Genomics, Aarhus University, Aarhus, Denmark

**Keywords:** Anxiolytics, Nonselective monoamine reuptake inhibitors, Receiver operating characteristic curve, Selective serotonin reuptake inhibitors, Treatment-resistant

## Abstract

**Background:**

Anxiety and depression are the most prevalent mental health disorders. The first-line treatment is antidepressants, such as serotonin reuptake inhibitors, but benzodiazepines and antihistamines are also used to treat anxiety. Only one-third of patients achieve remission with first-line treatment. Identifying responders and nonresponders to monotherapy prior to treatment could increase remission rates and reduce dropout. The aim of the current study was to predict response to antidepressants, benzodiazepines, and antihistamines from polygenic risk scores (PRSs) in individuals with anxiety and/or depression symptoms.

**Methods:**

We identified 2515 individuals in a genotyped cohort in the Swedish Twin Registry who had been prescribed drugs for anxiety and/or depression. Of these individuals, 1037 received monotherapy (555 with antidepressants, 169 with benzodiazepines, and 313 with antihistamines). The remaining 1478 individuals switched or added more drugs during the assessment period (2005–2018). The accuracy of 42 PRSs for psychiatric diagnoses as well as for nonclinical phenotypes in predicting mono- versus multitherapy was assessed using logistic regression.

**Results:**

Monotherapy with benzodiazepines was predicted by a PRS for depressive symptoms indexed by the Patient Health Questionnaire (odds ratio [OR] = 1.29), while monotherapy with antihistamines was predicted by a PRS for lifetime anxiety disorder (OR = 1.25) and a PRS for schizophrenia (OR = 1.24). None of the investigated PRSs significantly predicted monotherapy with antidepressants.

**Conclusions:**

Real-world data suggest that monotherapy with benzodiazepines or antihistamines can be predicted from PRSs related to anxiety, depression, and schizophrenia.

## Plain Language Summary

The study investigated how genetic factors measured by polygenic risk scores (PRSs) predict treatment response to antidepressants, benzodiazepines, and antihistamines for anxiety and depression. The researchers analyzed data from 2515 genotyped individuals in a large twin registry and linked the PRSs to single- and multiple-drug treatments. PRSs related to depression, anxiety, and schizophrenia predicted responses to benzodiazepines and antihistamines but not antidepressants. These findings suggest that genetic insights could help personalize treatments, thereby enhancing efficacy and reducing trial and error in prescribing.

Depressive and anxiety disorders are common psychiatric disorders worldwide. In 2019, 300 million people had a depressive disorder, and 301 million people had an anxiety disorder (1,2), and cases are increasing (2,3), as are prescriptions for antidepressants (4). Antidepressants, such as selective serotonin reuptake inhibitors, are the first-line treatment for depressive and anxiety disorders, but remission is only 30% to 35% for depressive disorders (5,6), with a somewhat better prognosis for anxiety disorders (5,7). Benzodiazepines are effective in treating anxiety disorders (8) but are considered a second-line treatment because of associated risks. Antihistamines have also been shown to be an effective treatment option for generalized anxiety disorder, offering an alternative to first-line treatments (9). Remission rate can be improved considerably by adding new drugs to a first-line treatment (10), but finding an effective treatment often takes a long time (11). Consequently, predicting who will benefit from a first-line treatment and who will need additional treatments could benefit patients and health care systems.

Studies that aim to predict treatment response have used clinical features and biological markers, such as polygenic risk scores (PRSs) (12). PRS prediction of treatment response in depressive disorders has yielded mixed results to date. A clear majority of published studies have used PRSs for major depressive disorder (MDD) to predict treatment response (13, 14, 15, 16, 17, 18, 19, 20, 21, 22), but PRSs for attention-deficit/hyperactivity disorder (ADHD), schizophrenia (SCZ), bipolar disorder, subjective well-being, openness, neuroticism, and general cognitive function (13, 14, 15, 16,19, 20, 21,23) have also been used. The largest of these studies used primary care records in the UK Biobank and Extended Cohort for E-health, Environment and DNA and reported a significant association between an ADHD-PRS and poorer antidepressant treatment response, defined as switching medication (13). Using genotyped cohorts from clinical studies, Amare *et al.* (23) found an association between the personality trait openness and better antidepressant treatment response. No other associations have been reported to survive corrected *p* levels, although tentative associations have been found between PRSs for MDD, SCZ, and neuroticism and treatment response (14,19,23).

Despite the growing body of research on PRS prediction of treatment response in MDD, there remains a notable gap in the literature regarding the association between response to pharmaceutical treatments and PRSs in individuals with anxiety disorders. Psychiatric disorders are known to show a considerable degree of genetic correlation. For example, MDD has a genetic correlation with SCZ, bipolar disorder, and ADHD of around 0.40 (24). However, the largest genetic overlap is found between MDD and anxiety disorders, with a genetic correlation of 0.80, suggesting a similar genetic background (24,25). The substantial genetic correlation that has been observed between anxiety and depression implies that genetic scores that predict treatment response for one disorder category are likely to be applicable to the other. Therefore, it is reasonable to investigate treatment response across these categories of disorders.

To fill the gap in the literature on PRS prediction of treatment response in anxiety, our aim was to predict treatment response to antidepressants, benzodiazepines, and antihistamines in anxiety and/or depression. Although antidepressants are recommended first-line treatment for depressive and anxiety disorders, benzodiazepines and antihistamines are often prescribed for symptoms of anxiety, and therefore they merit investigation. To our knowledge, PRS prediction of treatment response to benzodiazepines and antihistamines has not yet been investigated. Furthermore, because previous studies have only used few PRSs to predict treatment response, we wanted to test larger numbers of PRSs to examine whether PRSs for traits other than psychiatric disorders predict treatment response. In total, we used 42 different PRSs to predict treatment response in individuals with anxiety and/or depression symptoms. The study used data from genotyped individuals in the Swedish Twin Registry (STR), the records of which were linked to the National Prescribed Drug Register. Treatment response was defined as staying on a single drug over the assessment period, whereas nonresponse was defined as being prescribed multiple drugs.

## Methods and Materials

### Study Population

The current analysis is based on data from the population cohorts of Swedish twins who were born from 1959 to 1985 and 1986 to 1992 (STAGE [Study of Twin Adults: Genes and Environment] and YATSS [Young Adult Twins in Sweden Study]). They are part of the STR, which is one of the world’s largest databases of twin information and is used for research on the genetic and environmental factors that influence health and behavior (26,27). After saliva donation and initial quality control, which excluded all samples with call rates below 98%, the final sample included 11,210 individuals (STAGE: *n* = 8468, YATSS: *n* = 2742) (27). The genotyping for both cohorts was performed from 2017 to 2019 on the 650K Illumina Global Screening Array BeadChip.

### Measures

#### Anxiety and Depression Outcomes

The analyzed drugs were selected based on their regular use in treating anxiety and/or depressive disorders. Pharmaceutical prescription data for the study cohort from the Swedish Prescribed Drug Register was acquired through STR (28). They comprised a range of pharmacological classes identified by their Anatomical Therapeutic Chemical (ATC) codes (29):

• N06AA nonselective monoamine reuptake inhibitors
• N06AB selective serotonin reuptake inhibitors
• N06AF monoamine oxidase inhibitors (nonselective)
• N06AG monoamine oxidase A inhibitors
• N06AX other antidepressants
• N05BA benzodiazepine derivatives
• N05BE azaspirodecanedione derivatives
• R06A antihistamines for systemic use
• C07AA beta blocking agents (nonselective)
• N03AX other antiepileptics such as pregabalin

The prescription texts from the prescribing physician were searched using an algorithm that identified specific keywords linked to the ATC codes. Anxiety-related symptoms were defined by the keywords “worry,” “anxiety,” “anxiety issues,” and “panic,” indicating that medications were prescribed for anxiety. Depression-related symptoms were identified using the keywords “depression,” “depressive,” “dejection,” and “mood-enhancing,” indicating that medications were prescribed for depression.

Based on the outcomes of the algorithm, individuals were classified into 1 of 4 categories: 1) combined anxiety and depression symptoms, if the prescription text was linked to anxiety and depression symptoms; 2) anxiety only, if anxiety symptoms were linked without reference to depression; 3) depression only, if depression symptoms were linked without reference to anxiety; and 4) medication only, if the prescription text was not linked to either anxiety or depression.

In the dataset, each drug was sorted into separate columns for anxiety and depression based on prescription patterns. Individuals were coded as 1 if they received medication for anxiety and/or depression and 0 if they received medication without those keywords. Individuals without prescribed medication and with prescribed medication but without the keywords were excluded from analyses. All drug prescriptions were treated as lifetime occurrences, without distinction between individuals who received prescriptions for anxiety and depression concurrently or separately. This means that terms for anxiety and depression could appear together in the prescription text or in separate prescriptions.

#### Criteria for Drug Allocation and Duration of Use

In the next step, the data were filtered to identify individuals taking a single medication for depression symptoms, anxiety symptoms, or both combined (responders).

**Figure 1:**
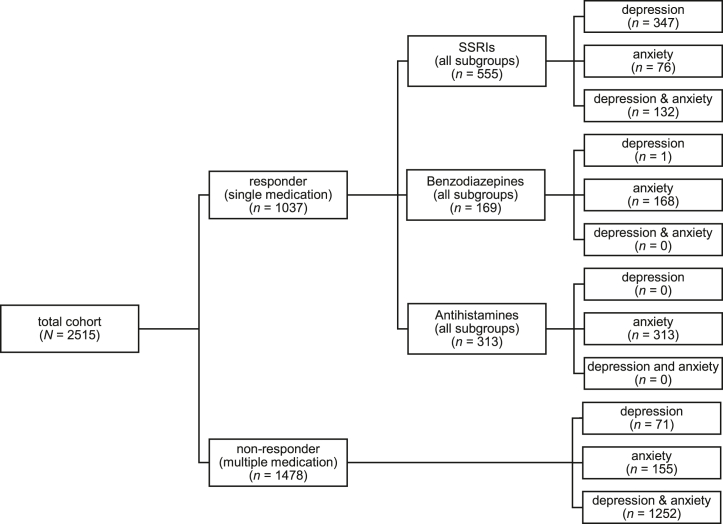
Medication allocation for depression and/or anxiety symptoms (*N* = 2515), divided into responders and nonresponders. SSRI, selective serotonin reuptake inhibitor.

Drugs that were predominately prescribed as a single treatment were grouped separately (antidepressants, benzodiazepines, antihistamines). Fourteen individuals were prescribed buspirone, propranolol, or pregabalin for anxiety symptoms. Due to the small sample size, they were excluded from statistical analyses. The other medications were included in the category in which individuals took more than 1 medication, which is used as a comparison group (nonresponders). This led to classification into more specific categories (Figure 1). Groups for single medications were coded as 0 (reference), and groups for multiple medications were coded as 1. In addition, the duration of medication use was calculated for each person based on the first and last prescription date. All other individuals who did not belong to one of those categories were excluded from the analyses, which resulted in a total of 2515 individuals.

#### Polygenic Risk Scores

A total of 42 PRSs were evaluated in this study; 36 of the 42 are from the Polygenic Index (PGI) repository, which aggregates scores derived from large-scale genome-wide association studies (GWASs) conducted in datasets such as 23andMe and the UK Biobank (30) (Table 1). Each phenotype has been associated with PRSs across 11 repository cohorts, including the STR, utilizing a leave-one-out approach to differentiate the discovery sample from the target cohorts (STAGE and YATSS). Consequently, PRSs in STAGE and YATSS were generated from GWAS summary statistics that exclude these particular datasets, thereby ensuring an unbiased targeting of phenotypes within the sample to evaluate the predictive efficacy of PRSs from the PGI repository. Detailed information regarding quality control and PRS construction methodologies has been reported previously (31).

**Table 1:** PRSs Included From the Polygenic Index in the Present Analysis

PRS Name	Abbreviation
Activity	Activity-PRS
Attention-Deficit/Hyperactivity Disorder	ADHD-PRS
Adventure	Adventure-PRS
Age First Birth	AFB-PRS
Asthma/Eczema/Rhinitis	ASTECZRHI-PRS
Asthma	Asthma-PRS
Alcohol Misuse	AUDIT-PRS
Body Mass Index	BMI-PRS
Cannabis	Cannabis-PRS
Cigarettes per Day	CPD-PRS
Cognitive Performance	CP-PRS
Depression	DEP-PRS
Drinks per Week	DPW-PRS
Educational Attainment	EA-PRS
Ever Smoke	Ever Smoke-PRS
Extraversion	EXTRA-PRS
Life Satisfaction: Family	FAMSAT-PRS
Life Satisfaction: Friends	FRIENDSAT-PRS
Hay Fever	Hay Fever-PRS
Height	Height-PRS
Highest Math	HIGHMATH-PRS
Left Out of Social Activity	LEFTOUT-PRS
Age First Menses	MENARCHE-PRS
Migraine	Migraine-PRS
Morning Person	MORNING-PRS
Narcissism	NARCIS-PRS
Nearsighted	Nearsighted-PRS
Number Ever Born Women	NEBwomen-PRS
Neuroticism	NEURO-PRS
Openness	OPEN-PRS
Childhood Reading	READING-PRS
Religious Attendance	RELIGATT-PRS
Risk	Risk-PRS
Self-Rated Health	SELFHEALTH-PRS
Self-Rated Math Ability	SELFMATH-PRS
Subjective Well-Being	SWB-PRS

PRS, polygenic risk score.

In addition to the 36 PRSs from the PGI repository, 6 PRSs were created based on diagnoses of lifetime anxiety disorder (LAD) (cases = 31,977, controls = 82,114) (32), MDD (cases = 170,756, controls = 329,443) (33,34), SCZ (cases = 67,390, controls = 94,015) (35), neuroticism scores (NEURO) (*n* = 323,415), the Generalized Anxiety Disorder 7-item scale (GAD-7) (*n* = 126,175), and the Patient Health Questionnaire-9 (PHQ-9) (*n* = 126,733). The NEURO-, GAD-7-, and PHQ-PRSs were all based on individual-level GWASs using data from the UK Biobank (30,36).

Exclusion criteria for the analyses were ambiguous single nucleotide polymorphisms (SNPs) (alleles A/T or C/G), trivial SNPs (showing no variation across the UK Biobank dataset), and SNPs with a minor allele frequency < 0.01.

For PRS construction, the LDAK software was used with the BLD-LDAK heritability model (37), in which the contribution of an SNP depends on minor allele frequency, linkage disequilibrium, and functional annotations. PRS construction was performed using LDAK-Bolt-Predict for individual-level data and LDAK-BayesR-SS for summary statistics, restricted to SNPs that overlap between cohort genotypes, the UK Biobank reference panel, and GWAS summary statistics. In total, 478,990 SNPs in STAGE and 487,169 SNPs in YATSS were used for PRS construction. Finally, all PRSs were standardized to *z* scores for interpretative purposes.

### Statistical Analyses

For each medication group, the number of participants was recorded according to the underlying symptoms and the average duration of prescription (absolute number, mean, interquartile range). *t* Tests were used to examine differences in average prescription length between the different medication groups.

To evaluate the predictive power of PRSs between monotherapies and multitherapy, logistic regression was performed using R (version 4.2.0) (38) and RStudio statistical software. Specifically, the packages questionr (39), pROC (40), and rms (41) were used.

Primary analyses of logistic regression included the respective monotherapies (antidepressants, benzodiazepines, antihistamines) in comparison to multitherapy including all symptom subgroups (Figure 1). The secondary analyses divided antidepressant treatment into symptom subgroups. Sex-specific sensitivity analyses were conducted for all drug groups to examine potential differences in PRS associations with treatment response between male and female participants. For each analysis, effect sizes were quantified as adjusted odds ratios (ORs) per SD of PRS, accompanied by corresponding 95% CIs for the best-performing PRS. An OR >1 indicated that a PRS was linked to multitherapy, while an OR <1 indicated a link to monotherapy. Pseudo-*R*^2^ (Nagelkerke *R*^2^) values were calculated for both the full model (incorporating all covariates including PRSs) and the baseline model (excluding PRSs). The baseline model included the variables sex, age, and the first 20 ancestral principal components (to control population stratification) as covariates. The proportion of variance attributable to the best-performing PRS was determined by calculating the difference between the 2 pseudo-*R*^2^ estimates (Δ*R*^2^).

The area under the receiver operating characteristic (ROC) curve was then calculated to compare the discriminative performance between the baseline model and the model with the best-performing PRS.

Subsequently, all PRSs were included in a single regression model to evaluate the predictive power of each score on outcomes while accounting for the influence of other PRSs. The change in *R*^2^ (Δ*R*^2^) was assessed to determine the extent to which the inclusion of all genetic indices within a single model improved the prediction of outcome variance.

All *p* values were corrected for multiple comparisons using the Benjamini-Hochberg procedure to control for the false discovery rate (FDR < 0.05) (42).

## Results

### Descriptive Information About the Medication Groups

A total of 2515 patients with depression and/or anxiety symptoms who were prescribed anxiolytic or antidepressant medications were included in the analysis. Table 2 reports the descriptive indices of the medication groups depending on the categorization from the prescription text as well as the average prescription length of single and multiple medication use. The *t* tests used to compare prescription length between the multiple and single medication groups indicated longer prescription length for multiple medication versus antidepressants (*t*_1705_ = 14.12, *p* < .001), multiple medication versus benzodiazepines (*t*_782_ = 22.71, *p* < .001), and multiple medication versus antihistamines (*t*_1780_ = 27.72, *p* < .001). The *t* tests conducted between single medication groups revealed a longer prescription length for antidepressants versus benzodiazepines (*t*_568_ = 8.28, *p* < .001) and antidepressants versus antihistamines (*t*_801_ = 10.99, *p* < .001). We also noted a distinct difference between medication groups regarding numbers of individuals with anxiety and/or depression. All but one individual prescribed monotherapy with benzodiazepines had an indication of anxiety. In the group prescribed monotherapy with antihistamines, all individuals had anxiety symptoms only (Table 2).

**Table 2:** Descriptive Information About the Medication Groups (*N* = 2515): Responders (Single Medication) vs. Nonresponders (Multiple Medications)

Medication		Number of Individuals			Average Duration in Days (IQR)
		Total	Male	Female	
Responders					
Antidepressants^a^	Depression	347	90	257	745 (651)
	Anxiety	76	20	56	544 (955)
	Depression and anxiety	132	41	91	1773 (2069)
	Total	555	151	404	1021 (1179)
Benzodiazepines^b^	Depression	1	0	1	1
	Anxiety	168	41	127	283 (105)
	Depression and anxiety	0	0	0	–
	Total	169	41	128	284 (104)
Antihistamines^c^	Depression	0	0	0	–
	Anxiety	313	82	231	204 (80)
	Depression and anxiety	0	0	0	–
	Total	313	82	231	204 (80)
Nonresponders					
Multiple Medication	Depression	71	20	51	1397 (1908)
	Anxiety	155	52	103	1479 (1862)
	Depression and anxiety	1252	318	934	2355 (3088)
	Total	1478	390	1088	1744 (2954)

IQR, interquartile range.

a Antidepressants: N06AB selective serotonin reuptake inhibitors, N06AA nonselective monoamine reuptake inhibitors, and N06AX other antidepressants.

b Benzodiazepines: N05BA benzodiazepine derivatives.

c Antihistamines: R06A antihistamines.

### PRS Prediction of Antidepressant Treatment Response

In the analysis focusing on the antidepressant treatment group, no significant association was observed between any of the investigated PRSs and treatment response after FDR correction (for full results, see Table S1). The ADHD-PRS yielded the strongest prediction and was significant at an uncorrected level, but not after FDR correction (*p* = .014; OR = 1.13; 95% CI, 1.03–1.25; *p*_corrected_ = .086), meaning that a higher ADHD-PRS was associated with multitherapy compared with monotherapy. However, after diagnosis was included as a covariate, the ADHD-PRS no longer remained the strongest predictor (*p* = .100; OR = 0.89; *p*_corrected_ = .268) (Table S2).

The baseline model accounted for 1.6% of the variance, while the inclusion of all PRSs resulted in 4.1% explained variance (Δ*R*^2^ = 2.5%).

The sensitivity analysis focused on symptom subgroups also revealed no significant associations between PRSs and treatment response (Table S1).

Sex-specific sensitivity analyses showed no significant results between PRSs and treatment response for males. However, a significant association with the depression PRS (DEP-PRS) was found for females (*p* = .004; OR = 1.20; *p*_corrected_ = .042) (Table S3).

### PRS Prediction of Benzodiazepine Treatment Response

In the examination of single PRSs and benzodiazepine usage for either anxiety or depression symptoms, a significant association was found between the PHQ-PRS and treatment response (*p* = .004; OR = 1.29; 95% CI, 1.09–1.54), with an FDR-corrected *p* value of .029 (Table S1), meaning that a higher PHQ-PRS was associated with multitherapy.

**Figure 2:**
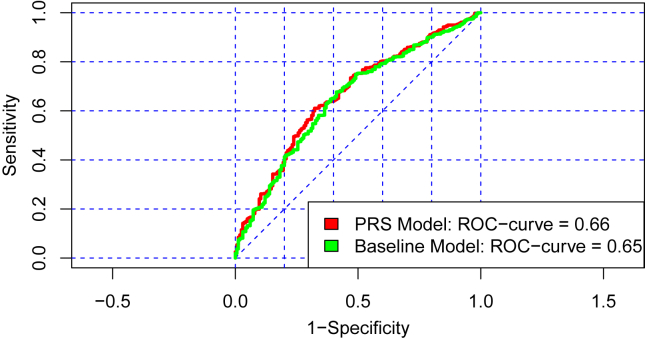
Comparison of ROC curves between the baseline model and the model with Patient Health Questionnaire-9 PRS inclusion (green = baseline model, red = model with PRSs). PRS, polygenic risk score; ROC, receiver operating characteristic.

The ROC curve value for the full model was 0.664, representing a 0.013 increase from the baseline model (Figure 2). The incremental increase in explained variance when the PHQ-PRS was included in the baseline model was modest (Δ*R*^2^ = 1.3%).

Because the benzodiazepine monotherapy group predominantly included individuals with anxiety, comorbidity of anxiety and depression could be a confounding factor. Therefore, we repeated the analysis, adding diagnosis as a covariate, which did not change the association with the PHQ-PRS (*p* = .005; OR = 1.28; *p*_corrected_ = .029) (Table S2).

Sex-specific sensitivity analyses showed no significant associations between PRSs and treatment response (Table S3).

The variance explained by the baseline model was 4.8%. With all PRSs included, the model fit increased to 11.7%.

### PRS Prediction of Antihistamine Treatment Response

The analysis focused exclusively on individuals prescribed antihistamines as monotherapy. The comparison group encompassed all individuals receiving multiple medications to ensure comparability with the antidepressant and benzodiazepine monotherapy groups.

**Figure 3:**
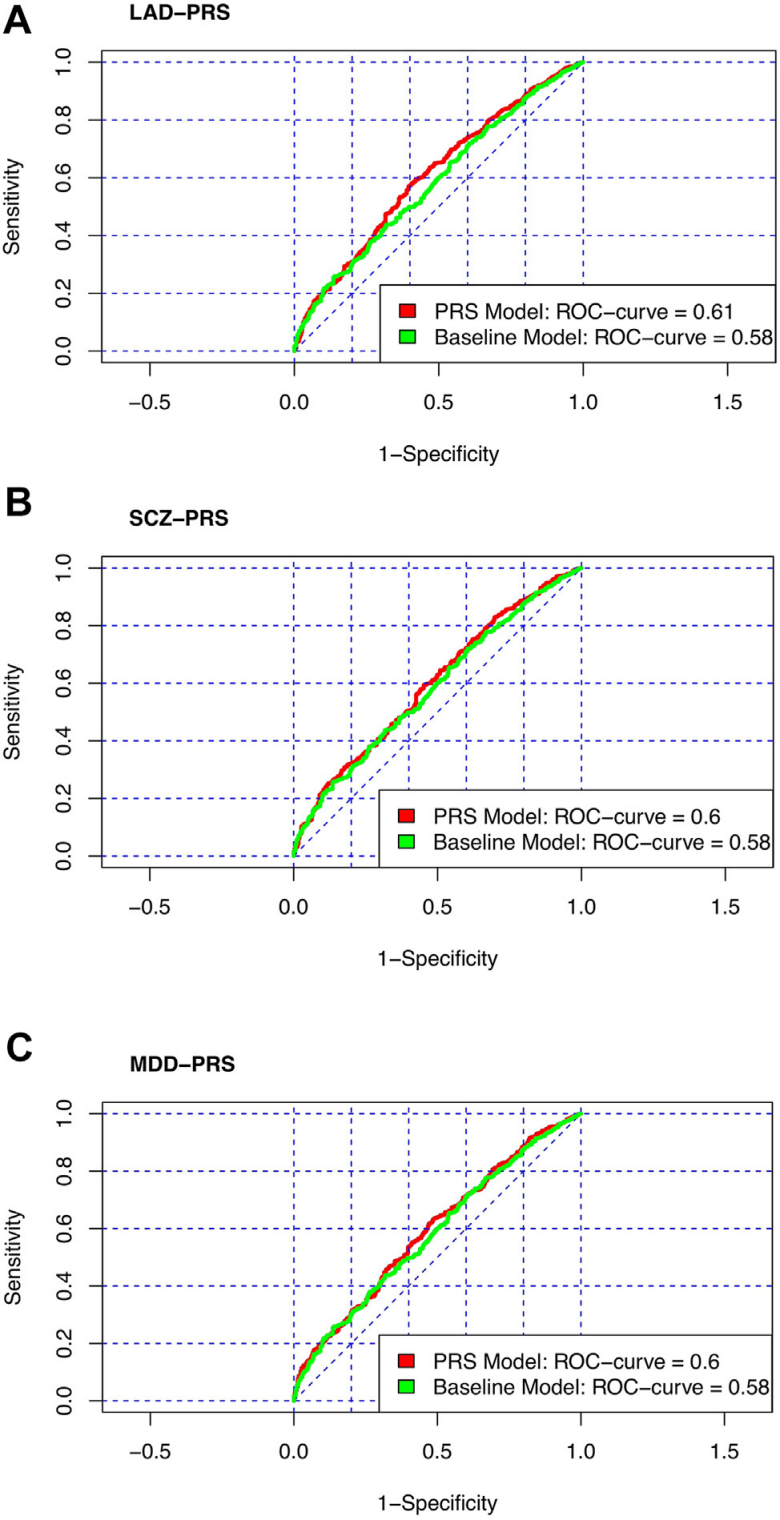
Comparison of the ROC curve between baseline model and model with PRSs: inclusion of LAD **(A)**, SCZ **(B)**, and MDD **(C)** PRSs (green = baseline model, red = model with PRSs). LAD, lifetime anxiety disorder; MDD, major depressive disorder; PRS, polygenic risk score; ROC, receiver operating characteristic; SCZ, schizophrenia.

The logistic regression analysis revealed significant associations between the LAD-PRS, the SCZ-PRS, and the MDD-PRS and treatment response, with greater associations between PRSs for multitherapy than monotherapy (Table S1). For the LAD-PRS, the analysis yielded a significant association (*p* = .001; OR = 1.25; 95% CI, 1.10–1.42), with an FDR-corrected *p* value of .007. The ROC curve was measured at 0.608, differing by 0.024 from the baseline model’s ROC (Figure 3A). There was a modest incremental increase in the explained variance when the LAD-PRS was included compared with the baseline model (Δ*R*^2^ = 1.1%). Concerning the SCZ-PRS, the analysis indicated a statistically significant association (*p* = .002; OR = 1.24; 95% CI, 1.08–1.41), with an FDR-corrected *p* value of .020. The ROC curve was 0.599, representing a 0.015 increase over the baseline model (Figure 3B). Moreover, the explained variance increased by approximately 0.9% when the SCZ-PRS was included in the baseline model. For the MDD-PRS, the analysis demonstrated a significant association (*p* = .004; OR = 1.21; 95% CI, 1.06–1.37), with an FDR-corrected *p* value of .046. The ROC curve was 0.596, with a difference from the baseline model of 0.012 (Figure 3C). There was a modest rise in the explained variance observed when the MDD-PRS was incorporated into the baseline model (Δ*R*^2^ = 0.7%).

Because the antihistamine monotherapy group exclusively included individuals with anxiety, comorbidity of anxiety and depression could be a confounding factor. Therefore, we repeated the analyses, adding diagnosis as a covariate. After FDR correction, significant associations were observed for the SCZ-PRS (*p* = .001; OR = 1.26; *p*_corrected_ = .0008) and the LAD-PRS (*p* = .001; OR = 1.23; *p*_corrected_ = .011); however, no significant association was found for the MDD-PRS (*p* = .009; OR = 1.18; *p*_corrected_ = .096) (Table S2).

Sex-specific sensitivity analyses revealed no significant associations between PRSs and treatment response for males. In contrast, a significant association between the LAD-PRS and treatment response was observed for females (*p* = .001; OR = 1.28; *p*_corrected_ = .019) (Table S3).

The baseline model accounted for 2.6% of the variance. With the integration of all PRSs, the explained variance increased to 8.4% (Δ*R*^2^ = 5.8%).

### Comparison of the PHQ-PRS, LAD-PRS, SCZ-PRS, and MDD-PRS Between the Different Drugs

**Figure 4:**
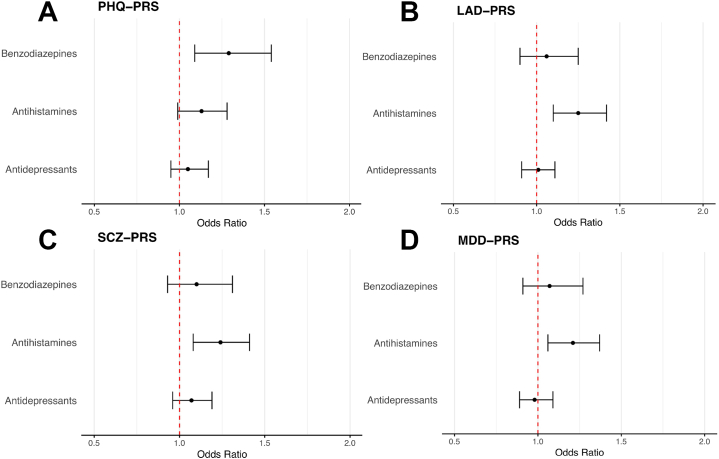
Odds ratios and 95% CIs for the PHQ-PRS **(A)**, LAD-PRS **(B)**, SCZ-PRS **(C)**, and MDD-PRS **(D)** across antidepressants, benzodiazepines, and antihistamines. LAD, lifetime anxiety disorder; MDD, major depressive disorder; PHQ, Patient Health Questionnaire; PRS, polygenic risk score; SCZ, schizophrenia.

Figure 4 illustrates the ORs and 95% CIs before FDR correction for the PHQ-PRS, LAD-PRS, SCZ-PRS, and MDD-PRS across the 3 drug treatment groups. The figure shows a clear pattern, with a trend toward ORs > 1 for all PRSs in both the benzodiazepine and antihistamine groups. In the antidepressant group, the PRSs showed a more consistent pattern around the value 1, with no discernible trend. For the SCZ-PRS and the PHQ-PRS, a trend toward higher ORs across all treatment groups was observed.

## Discussion

We investigated associations between 42 PRSs and treatment response to antidepressants, benzodiazepines, or antihistamines prescribed for anxiety and/or depression symptoms. PRSs related to anxiety (LAD-PRS), depression (PHQ-PRS), and schizophrenia (SCZ-PRS) predicted treatment response to benzodiazepines and antihistamines. Treatment response to antidepressants could not be predicted from the PRSs in our sample.

In the benzodiazepine treatment group, a higher PHQ-PRS predicted worse treatment response. The PHQ-PRS reflects self-reported depression symptoms. To our knowledge, no previous studies have examined PRS prediction of benzodiazepine treatment response. Like many drugs, benzodiazepines are metabolized by CYP (cytochrome P450) enzymes. While genetic variants in *CYP2C19* and *CYP2B6* have been investigated for effects on drug safety (43), reports on their effect on treatment response are lacking. In general, monotherapy with benzodiazepines is considered effective for anxiety, with rapid onset and high tolerance (8,44). Also, nearly half of all patients who do not respond to antidepressants are prescribed benzodiazepines (45), showing that they could be an effective adjunct therapy. However, when prescribing benzodiazepines, physicians generally consider that patients may develop addiction or have other vulnerability factors that argue against prescribing. We note that individuals in our study who were prescribed benzodiazepine monotherapy did not have comorbid anxiety and depression. Furthermore, the duration for which they were prescribed medication was significantly shorter than that for the groups prescribed antidepressants or multitherapy. Therefore, the finding that the PHQ-PRS was lower in this group than in the multitherapy group could be driven by lower symptom severity rather than by drug efficacy. We included diagnosis as a covariate, which resulted in the same significant association being found between treatment response and the PHQ-PRS. This shows that comorbidity does not explain why the PHQ-PRS predicted monotherapy with benzodiazepines. Future studies should stratify groups based on symptom severity and comorbidity in large samples to test whether a PHQ-PRS predicts treatment response to benzodiazepines.

The group prescribed antihistamines as monotherapy had significantly lower PRSs for MDD, SCZ, and LAD than the group prescribed multitherapy. These associations could indicate that a higher genetic liability for these conditions is associated with lower efficacy of antihistamine treatment. Although antihistamines are considered effective against anxiety (46), to our knowledge no study has investigated genomic predictors of treatment response. However, the antihistamine group also did not include any individuals with comorbid anxiety and depression or depression only, suggesting comorbidity as a confounding factor. Therefore, we included diagnosis as a covariate in our analyses. The associations of the SCZ-PRS and the LAD-PRS with antihistamine treatment remained significant, while the association with the MDD-PRS was no longer significant. This shows that the SCZ-PRS and the LAD-PRS predicted monotherapy with antihistamines even when controlling for comorbidity in the multitherapy group.

Sex-specific analyses showed a significant association between DEP-PRS and antidepressant treatment response in women, consistent with previous studies that have linked genetic liability for depression to treatment resistance across sexes (13, 14, 15, 16, 17, 18, 19, 20, 21, 22). In contrast, no significant results were observed for men in our sample, suggesting a possible sex difference in antidepressant treatment resistance. However, the smaller sample size for the male group might have limited our ability to detect significant results. Further research with larger samples is needed to confirm these findings. Likewise, the lack of significant sex-specific results in the antihistamine (except the LAD-PRS) and benzodiazepine groups is probably due to smaller sample sizes and reduced statistical power.

When we compared PRS prediction of treatment response across the different medication groups, several noteworthy trends and potential implications emerged. In the antihistamine and benzodiazepine monotherapy groups, PRSs for PHQ, LAD, SCZ, and MDD predicted treatment response in the same direction with similar effect sizes. The shared sedative effects of benzodiazepines and antihistamines may support these similar results (16,47). In contrast, the antidepressant group exhibited smaller effect sizes for the same PRSs (Figure 4). This dissimilarity could be due to the more heterogeneous nature of the group of individuals who comprised the antidepressant monotherapy group, which included individuals with anxiety and/or depression symptoms. Compared with benzodiazepines and antihistamines, antidepressants do not have sedative effects (48), which may also explain the different patterns that we observed across monotherapy groups.

There are currently few GWASs that have focused on treatment response to antidepressant and anxiolytic drugs. Two studies have computed PRSs for treatment response to antidepressants (19,22) based on a GWAS of treatment response, but the numbers of responders and nonresponders in these studies were too small to yield reliable PRSs that could be used for out-of-sample prediction. Future studies could use large datasets, such as the UK Biobank or FinnGen, to obtain more accurate predictors of treatment response. Alternative approaches that use whole-genome sequencing could, for example, inform about how copy number variations [e.g., (49)], including variable number of tandem repeats, predict treatment response to antidepressants and anxiolytics in future studies. Using multiomics data to predict treatment response is another approach that holds promise for increasing prediction accuracy [e.g., (50,51)].

The current study has several limitations. First, the definition of treatment response and nonresponse relied on medication patterns rather than clinical assessments. This might have biased our results, although we note that the response rate in our sample is consistent with that in previous studies that used clinical assessment. Moreover, prescription data were only obtained for a limited period of time, meaning that some people might have been prescribed other drugs before the start of the period during which prescriptions were registered. This might have inflated the numbers of individuals categorized as being prescribed monotherapy in our study. Additionally, participants were categorized according to their symptoms as judged by the physician who prescribed the drugs rather than from ICD diagnoses. However, in a previous study, we showed that PRS prediction of disorder categories from ICD diagnosis was almost identical to prescription text classification (52), indicating that our innovative use of text analysis in the National Prescribed Drug Register is valid. Furthermore, a natural limitation of this study is that PRSs are not comprehensive because they account for only a small proportion of the genetic factors involved. If PRSs were more accurate, we would expect to see an increase in ORs.

### Conclusions

The current study identified 3 significant associations between PRSs (PHQ, LAD, SCZ) and treatment response to benzodiazepines and antihistamines in individuals with anxiety symptoms. The results show that PRSs based on a GWAS of psychiatric diagnosis can predict treatment response using real-world data.

## Supplementary Material

Tables S1–S3

Key Resources Table
